# Social Tobacco Warnings Can Influence Implicit Associations and Explicit Cognitions

**DOI:** 10.3389/fpsyg.2019.00324

**Published:** 2019-02-26

**Authors:** Barbara C. N. Müller, Rinske Haverkamp, Silvia Kanters, Huriye Yaldiz, Shuang Li

**Affiliations:** ^1^Behavioural Science Institute, Radboud University, Nijmegen, Netherlands; ^2^Radboud UMC, Nijmegen, Netherlands

**Keywords:** tobacco warning labels, smoking, risk perception, attitudes toward smoking, implicit associations

## Abstract

Previous research showed that fear-inducing graphic warning labels can lead to cognitive dissonance and defensive responses. Less threatening, social-related warning labels do not elicit these defensive responses, making them more effective in preventing smoking in adults. Given that smoking numbers are still too high among youngsters, it is crucial to investigate how warning labels should be designed to prevent teenagers from starting smoking in the first place. In two studies, we investigated whether comparable effects of social-related warning labels could be observed in a group of teenagers (14–17 years) who are not yet legally allowed to smoke. In addition, we tried to replicate earlier findings with smoking and non-smoking adults. Participants were presented with either health warning labels, social warning labels, or no warning labels. Subsequently, their explicit cognitions (i.e., risk perception, attitude toward smoking) and their implicit associations of smoking with healthiness/unhealthiness (Study 1a and Study 1b) and with positivity/negativity (Study 2a and Study 2b) were assessed. Results showed that in both studies, adult smokers had a higher risk perception and a more positive attitude toward smoking than adult non-smokers. Additionally, social warning labels lead to stronger implicit associations between smoking and negativity in Study 2 in the adult groups. In the teenage group, social warning labels lead to more positive attitudes than health warning labels in Study 2. No further effects on risk perception or implicit associations were found in the teenage group. Possible explanations are discussed.

## Introduction

By now, it is widely known that smoking is the main cause for deadly diseases such as cancer, COPD, and cardiovascular diseases (Laniado-Laborín, 2009; Centers for Disease Control and Prevention et al., 2010; Furrukh, 2013). Nevertheless, 25% of the population in the EU aged 15 and older need several moments a day to smoke a cigarette or other tobacco products (Agafitei and Hrkal, 2016). As a result, in 2016, approximately 1.4 million people in Europe died as a consequence of consuming tobacco products (see also Cahn et al., 2008). To reduce the immense costs related to smoking diseases and to increase the overall health of the population, governments try to discourage people from smoking. For example, warning labels on cigarette packages are developed, which have the goal to inform smokers of the negative consequences of smoking and encourage them to stop. Since May 2016, the layout of the cigarette packages has changed drastically in the whole European Union (see also Hammond, 2001), with warning labels with sentences such as “Smoking kills” supplemented by images of somatic consequences of smoking, such as a black lung or a dying man, and comparable graphical warning labels are planned in the United States. These images aim to increase fear, disgust, and aversion against smoking (Hammond et al., 2006; Glock et al., 2013a,b), which should result in fewer people who start smoking and more cessation attempts among smokers (Hammond et al., 2006). Nevertheless, smoking initiation is highest among young adults around 18 years (Marcon et al., 2018). Therefore, the current research aims to investigate how warning labels can be made more effective and focuses on whether and how the content of the warning labels influences teenagers’ implicit associations and explicit cognitions toward smoking.

Warning labels on cigarette packages have the aim to inform people, especially smokers, of the negative consequences of smoking and to increase their risk perception, that is, their perception of how likely it is that they will suffer from the negative health consequences caused by smoking (Strahan et al., 2002). The currently used health warning labels have been shown to induce threat (Hansen et al., 2010), by using so-called fear appeals. Fear appeals are persuasive messages that induce fear and should thereby lead to self-protective actions and behavior change (Rogers, 1983; Witte and Allen, 2000). However, these fear appeals can also lead to cognitive dissonance (Hansen et al., 2010), a mental state of discomfort that occurs when someone holds two psychologically inconsistent cognitions at the same time (Festinger, 1957). For example, this can be elicited when a person smokes but at the same time is well aware of the fact that smoking is bad for ones’ health. This cognitive dissonance leads to negative feelings (Harmon-Jones, 2001). To resolve the feeling of cognitive dissonance, people can either be more motivated to change their behavior, resulting in a reduction of their smoking behavior (Hansen et al., 2010), or in changing their cognitions in favor of smoking, resulting in continuing to smoke.

Correlational studies demonstrated that warning labels can lower the appeal of the package, and create higher levels of negative affect such as anxiety (Kees et al., 2006; Mannocci et al., 2012; Layoun et al., 2017). Furthermore, audiences are much more likely to pay attention to messages with graphic images than those with text only (O’Hegarty et al., 2006; Layoun et al., 2017). The graphic images increase the negative thoughts and feelings about smoking (Hammond et al., 2003), and smokers’ perceived intention of quitting smoking (Kees et al., 2006), which suggest that they can be effective in enhancing smoking-related risk perception (O’Hegarty et al., 2006; Layoun et al., 2017). This higher risk perception suggests that the participants are more aware of the negative consequences of smoking after having seen the graphic warning labels. Thus, text-plus-graphic warning labels represent an opportunity to decrease smoking behavior. However, experimental studies have not been able to provide much evidence for the usefulness of warning labels and the results are inconsistent (for an overview see Kok et al., 2017). Although the purpose of health warning labels is to induce fear and motivate people to stop smoking, fear can lead to unintended side-effects such as defensive responses and boomerang effects due to cognitive dissonance (Glock and Kneer, 2009; Glock et al., 2013a; Peters et al., 2013). Thereby, the stronger the fear appeal is, the more likely it is that defensive responses are induced (De Meyrick, 2001), which help to protect an individual against the threat (i.e., feeling fear) and maintain a positive self-image (Ruiter and Kok, 2005). For example, research has shown that smokers who are exposed to visual warning labels can react in a defensive way in which they minimize the probability that they will suffer from the diseases represented in the warning labels (Harris et al., 2007). This, in turn, reduces the intention to change the target behavior (Good and Abrahams, 2007).

Next to cognitive dissonance, health warning labels can also result in psychological reactance, a motivational state which appears when an individual’s freedom to choose is threatened. He or she will then be motivated to restore this freedom, for example by doing the exact opposite of what is asked (Brehm, 1996). It has been shown that smokers who were exposed to graphic warning labels were much more likely to experience higher levels of reactance (Erceg-Hurn and Steed, 2011). This reactance might lead to an increase in smoking (Glock et al., 2012). It has been shown that, compared to health warning labels using fear appeals, anti-tobacco advertisements without fear-inducing images cause viewers to spend more time encoding the messages (Süssenbach et al., 2013). Furthermore, warning labels formulated as questions (Glock et al., 2013b) or labels which contradict common smoking-related positive outcome expectancies (e.g., smoking reduces stress), do not elicit defensive responses, and thus result in higher smoking-related risk perception (Glock et al., 2013b). It is further supported by research that less aversive warning labels do not induce threat, and therefore no defensive responses are elicited, which makes them more effective than fear-inducing warning labels [Süssenbach et al., 2013; but see van Dessel et al. (2018) for null findings of graphical warning labels].

In the current research, we aim to test whether these positive effects can be replicated in a younger target group. As most smokers have started smoking by the age of 18 (Marcon et al., 2018), the effectiveness of tobacco warning labels could be largely increased by additionally focusing on teenagers and prevent them from starting to smoke. Teenagers are very sensitive to negative social consequences and peer pressure (Glock et al., 2013a; Liao et al., 2013). Therefore, warning labels challenging positive outcome expectancies as used in earlier studies (Glock et al., 2013b) could have even more positive effects in teenagers. Next to teenagers, we tested whether earlier findings could be replicated in an adult smoker and an adult non-smoker group. Four studies were conducted in which participants were randomly presented with either currently used health warning labels or social warning labels challenging positive outcome expectancies, or not presented with any warning label. Subsequently, both explicit cognitions (i.e., risk perception and attitudes toward smoking) and implicit associations assessed with the Single Target Implicit Association Task (ST-IAT) were measured. We focused on both explicit and implicit processes because studies have shown that explicit attitudes and implicit associations can be influenced differently by anti-smoking messages (Smith and de Houwer, 2015), and both are important predictors of addictive behavior such as cigarette smoking (Wiers and Stacy, 2006). We expected that social warning labels would lead to more negative explicit cognitions toward smoking and stronger implicit negative associations and smoking in all groups. These effects were expected to be the strongest in the teenage group^1^.

## Study 1A

### Methods

#### Participants and Design

Using G^∗^Power (Faul et al., 2007), *a priori* estimation of statistical power of (1 – β) = 0.8 and an estimated effect size of $\eta_{p}^{2}$ = 0.05 [derived from Glock et al. (2013a)] lead to a minimum sample size of 31 participants per group for the implicit measure of the experiment. Based on an *a priori* estimation of statistical power of (1 – β) = 0.8 and an estimated effect size of $\eta_{p}^{2}$ = 0.07 [derived from Glock et al. (2013b)], a minimum of 22 participants per group were required for the explicit measures of the experiment^2^. One-hundred-sixty-one students from Radboud University (73 smokers and 88 non-smokers; 31 males, 127 females, 3 missing values on gender) with an age range between 18 and 33 years old (*M* = 20.21, *SD* = 2.39) participated in this study. A 3 (warning label condition: health warning label vs. social warning label vs. control) × 2 (group: smokers vs. non-smokers) between-subjects design was used, with risk perception, explicit attitude toward smoking, compensatory health beliefs, and implicit associations toward smoking as dependent variables. Participants were randomly assigned to the warning label conditions using Inquisit 4 (Inquisit computer software, 2015). Distribution to conditions was as follows: 50 participants in the health warning label condition (21 smokers, 29 non-smokers), 57 participants in the social warning label condition (27 smokers, 30 non-smokers), and 54 participants in the control condition (25 smokers, 29 non-smokers). Via an online participant pool from the university, participants signed up for the study, and received a link to download the study file onto their computer. Before participants could start with the experiment, they were asked to ensure that they would not be interrupted for the duration of the experiment (approximately 20 min), and had to give active consent. The study was conducted according to the principles expressed in the Declarations of Helsinki and according to the guidelines of the institutional review board (Ethics Committee Faculty of Social Sciences, Radboud University, Nijmegen, Netherlands). Ethical approval was at the time of data collection not required by the Institution’s guidelines and national regulations, as the research was not of a medical nature and there were no potential risks to the participants.

#### Procedure and Materials

Participants were instructed to perform a categorization task and complete several short questionnaires. In the health warning label condition, participants were asked to read cigarette warning labels with fear-inducing messages referring to the negative health consequences of smoking. These labels are currently used in Belgium. In the social warning label condition, participants were asked to read cigarette warning labels with negative social consequences of smoking. The layout of all labels was kept similar to the existing warning labels on cigarette packages, and both types of labels contained text and matched images. Both groups were shown eight labels, with each label displayed for 5 s in the middle of the screen. No packages and warning labels were presented to participants in the control condition and participants immediately started with the following task. We decided to use a control condition in which no stimuli were presented to be able to measure baseline explicit and implicit cognitions, i.e., without any influence of for example text that is present on textual warning labels. Subsequently, participants were asked to perform the categorization task, a ST-IAT used in earlier research (see Glock et al., 2013a). They were instructed to categorize the upcoming images as smoking related, healthy, or unhealthy by pressing the “E” and the “I” keys as quickly as possible, without making too many errors. Three different image categories were used: (1) smoking-related images, in which people in daily situations with a cigarette were displayed, (2) healthy images displaying healthy food (e.g., fruits), beverages (e.g., water), and activities (e.g., walking), and (3) unhealthy images displaying unhealthy foods like burgers, beverages like alcohol, and unhealthy activities such as watching TV and eating snacks (for examples see Glock et al., 2013a). Participants first underwent 20 practice trials in which they had only to categorize healthy and unhealthy images. Next, smoking pictures were included, with healthy pictures and smoking pictures sharing the same key (incompatible block). Participants completed 20 practice trials and 40 critical trials. In the following block, smoking pictures were paired with unhealthy pictures (compatible block). Again, participants completed 20 practice trials and 40 critical trials. Half of the participants received the compatible trials first, and the other half received the incompatible trials first. The use of “E” and “I” keys was also counterbalanced across participants.

After completing the ST-IAT (Guttman split-half = 0.74), participants’ risk perception, explicit attitude toward smoking, and their compensatory health beliefs were assessed. Firstly, participants had to answer six items about risk perception on a five-point Likert scale (ranging from 1 “totally not likely” to 7 “very likely”), indicating how likely it is that they get a smoking-related disease (e.g., “How likely will you get a heart attack?”; Cronbach’s α = 0.937; Glock et al., 2013b). Secondly, they had to answer seven items about their explicit attitude toward smoking on a five-point Likert scale (ranging from 1 “totally not possible” to 7 “very possible”), indicating how much they agreed with the items (e.g., “Smoking helps to concentrate”; Cronbach’s α = 0.927). Thirdly, they had to answer seventeen items about their compensatory health beliefs on a five-point Likert scale (ranging from 1 “totally not possible” to 7 “very possible”), indicating how likely it is that negative consequences of an unhealthy behavior (e.g., smoking) could be compensated for by performing another healthy behavior (e.g., eating healthy; “Smoking from time to time is OK if you eat healthy.”; Spearman-Brown Coefficient = 0.634; Glock et al., 2013a)^3^.

In the end, participants completed a collection of demographic questions asking about gender, age, their smoking habits, and what they think of the aim and hypotheses of the study. After completion of the questionnaire, they were thanked for participation and informed how to contact the experimenter in case they would like to know more about the aim, hypotheses, and results of the study.

### Results and Discussion

Data were analyzed with SPSS 24. Mean scores for risk perception and attitude were calculated. For compensatory health beliefs, only the two smoking-related items were included in the analysis. For the implicit associations measured by the ST-IAT, D-scores based on the improved scoring algorithm as described in Greenwald et al. (2003) were calculated. A higher ST-IAT D-score indicates more positive associations with smoking. For all means and standard deviation, see Table 1.

**Table 1:** Means, standard deviation for all dependent variables of Study 1a.

		Risk perception	Explicit attitude	Compensatory health beliefs	Implicit associations
Non-smokers	Social warning condition	*M* = 2.46, *SD* = 1.47	*M* = 1.92, *SD* = 1.07	*M* = 1.77, *SD* = 0.94	*M* = –0.39, *SD* = 0.30
	Health warning condition	*M* = 3.01, *SD* = 1.32	*M* = 1.67, *SD* = 0.77	*M* = 1.89, *SD* = 0.87	*M* = –0.35, *SD* = 0.48
	Control condition	*M* = 2.81, *SD* = 1.26	*M* = 1.76, *SD* = 1.07	*M* = 1.74, *SD* = 1.11	*M* = –0.36, *SD* = 0.34
	Overall	*M* = 2.76, *SD* = 1.36	*M* = 1.78, *SD* = 0.97	*M* = 1.80, *SD* = 0.96	*M* = –0.37, *SD* = 0.38
Smokers	Social warning condition	*M* = 3.49, *SD* = 1.29	*M* = 3.67, *SD* = 1.27	*M* = 2.59, *SD* = 1.43	*M* = –0.31, *SD* = 0.48
	Health warning condition	*M* = 4.50, *SD* = 0.98	*M* = 3.99, *SD* = 0.92	*M* = 3.12, *SD* = 1.24	*M* = –0.39, *SD* = 0.30
	Control condition	*M* = 3.52, *SD* = 1.52	*M* = 3.25, *SD* = 1.42	*M* = 2.62, *SD* = 1.09	*M* = –0.27, *SD* = 0.35
	Overall	*M* = 3.79, *SD* = 1.36	*M* = 3.62, *SD* = 1.25	*M* = 2.75, *SD* = 1.28	*M* = –0.32, *SD* = 0.38

#### Risk Perception, Attitude Toward Smoking, and Compensatory Health Beliefs

A MANOVA with warning label condition (health warning label vs. social warning label vs. control) and group (smokers vs. non-smokers) as between-subjects factor, and the mean scores of the explicit attitude toward smoking, risk perception, and compensatory health beliefs as dependent variables was conducted. For risk perception, a significant main effect of warning label condition was found, *F*(2,155) = 4.780, *p* = 0.010, $\eta_{p}^{2}$ = 0.058, with participants in the health warnings condition scoring higher on risk perception (*M* = 3.64, *SD* = 1.40) than participants in the social warnings condition (*M* = 2.95, *SD* = 1.47; *p* < 0.010, *d* = 0.48). The remaining comparisons were non-significant (health warnings condition vs. control condition *p* = 0.079, *d* = 0.35; social warnings conditions vs. control condition *p* = 1.00, *d* = 0.13). Furthermore, a significant main effect of group was found, *F*(1,155) = 25.960, *p* < 0.001 $\eta_{p}^{2}$ = 0.143, with smokers scoring higher on risk perception (*M* = 3.80, *SD* = 1.36) than non-smokers (*M* = 2.76, *SD* = 1.36). The interaction between warning label condition and group was not significant, *F*(2,155) = 1.099, *p* = 0.336, $\eta_{p}^{2}$ = 0.014.

For attitude, the effect of warning label condition was not significant, *F*(2,155) = 1.399, *p* = 0.250, $\eta_{p}^{2}$ = 0.018. However, a significant main effect of group was found, *F*(1,155) = 112.093, *p <* 0.001, $\eta_{p}^{2}$ = 0.420, with smokers having a more positive attitude toward smoking (*M* = 3.62, *SD* = 1.26) than non-smokers (*M* = 1.78, *SD* = 0.97). The interaction between warning label condition and group was not significant, *F*(2,155) = 1.866, *p* = 0.158, $\eta_{p}^{2}$ = 0.024.

For compensatory health beliefs, no significant effect of warning label condition was found, *F*(2,155) = 1.447, *p* = 0.238, $\eta_{p}^{2}$ = 0.018. However, the significant main effect of group was found, *F*(1,155) = 30.087, *p <* 0.001, $\eta_{p}^{2}$ = 0.163, with smokers scoring higher on compensatory health beliefs (*M* = 2.76, *SD* = 1.28) than non-smokers (*M* = 1.80, *SD* = 0.96). The interaction between warning label condition and group was not significant, *F*(2,155) = 0.471, *p* = 0.625, $\eta_{p}^{2}$ = 0.006.

#### Implicit Associations

An ANOVA with warning label condition (health warning label vs. social warning label vs. control) and group (smokers vs. non-smokers) as between-subjects factors, and the D-score as dependent variable showed no significant differences between the three warning label conditions, *F*(2,155) = 0.192, *p* = 0.825, $\eta_{p}^{2}$ = 0.002, no significant differences between the two groups, *F*(1,155) = 0.432, *p* = 0.512, $\eta_{p}^{2}$ = 0.003, and no significant interaction between warning label condition and group, *F*(2,155) = 0.565, *p* = 0.570, $\eta_{p}^{2}$ = 0.007.

Not surprisingly, our results show that smokers have a higher risk-perception, a more positive attitude toward smoking, and stronger compensatory health beliefs than non-smokers. In addition, participants who watched the health warning labels scored higher on risk-perception than participants who watched the social warning labels, independent of smoking status. Interestingly, no significant increase in defensive responses, such as a more positive attitude toward smoking after health warning exposure, was observed, which is not in line with earlier research findings (Süssenbach et al., 2013). Furthermore, no significant difference on implicit measures was observed. These null findings might be a lack of power in the adult smoker group. However, very recent work comparing graphical warning labels with text-only warning labels with a much larger sample (*N* = 7757) also found no significant effects on implicit associations measured with an IAT, and no effects on explicit evaluations (van Dessel et al., 2018) in an adult population of smokers, occasional smokers, and non-smokers. Our null-findings are in line with this work.

In Study 1b, we collected data in a teenage sample aged from 14 to 17, to investigate how they respond to the different types of warning labels. Note that this sample is not yet allowed to smoke in the Netherlands. Investigating the influence of warning labels in this group is important for several reasons: Teenagers are very prone to evaluations from their peers and sensitive to negative feedback (Liao et al., 2013). Therefore, it might be that social related negative consequences have a stronger impact on them than health warnings. In addition, next to motivate smokers to stop smoking, warning labels are also designed to also discourage teenagers from starting to smoke, but it is still unknown that whether this is actually the case and whether the design could be improved.

## Study 1B

### Methods

#### Participants and Design

One-hundred-and-five students from a Dutch secondary school participated in this study. Thirteen students (4 participants from the health warning label condition, 3 participants from the social warning label condition, 6 participants from the control condition) were excluded because they smoked, and two participants were excluded because responses indicated that they did not participate seriously (i.e., stating that their native language was ancient Latin; that they are a student at the University of Leiden). Thus, the final sample consisted of 90 students (36 males, 53 females, 1 missing value on gender) with an age range between 14 and 17 years old (*M* = 15.39, *SD* = 0.70). Students were studying at VWO level (*N* = 50) and HAVO level (*N* = 40). Distribution to conditions was as follows: 28 participants in the health warning label condition, 33 participants in the social warnings label condition, and 29 participants in the control condition. Active informed consent was acquired from the director of the school, and subsequently, passive informed consent was acquired from the parents or primary caretakers of the participating students. Before participating in the study, all students gave active written informed consent by themselves.

A single factor (warning label condition: health warning label vs. social warning label vs. control) between-subject design was used, with risk perception, explicit attitude toward smoking, and implicit associations toward smoking as dependent variables. Participants were randomly assigned to one of the three conditions using Inquisit 4 (Inquisit computer software, 2015). The study was conducted according to the principles expressed in the Declarations of Helsinki and according to the guidelines of the institutional review board (Ethics Committee Faculty of Social Sciences, Radboud University, Nijmegen, Netherlands). Ethical approval was at the time of data collection not required by the Institution’s guidelines and national regulations, as the research was not of a medical nature and there were no potential risks to the participants.

#### Procedure and Materials

On the days of the experiment, students were picked up from their classes in groups of 9 students. Before entering the room where the experiment took place, the students got verbal instructions. They were told to choose a computer and follow the instructions displayed on the computer screen. If they had any question, they could ask during the experiment before starting the following block. They were also informed that they could stop whenever they wanted to. After the verbal instructions, students read and signed the active informed consent before entering the room. After everyone sat down by a computer of their choice, the experimenter gave a sign to start the experiment. Students were not allowed to speak to each other during the experiment.

The same materials were used as in Study 1a. After the presentation of the warning labels, participants first performed the ST- IAT (Guttman split-half = 0.71), and subsequently the questionnaires measuring smoking-related risk perception (Cronbach’s α = 0.93) and attitudes toward smoking (Cronbach’s α = 0.90). One item of risk perception (COPD) was not included in the student sample as it was unclear whether students already knew about this disease. The last part of the questionnaire contained demographic questions asking about gender, age, grade, school level, their smoking status, and smoking habits.

Students who completed the experiment had to wait until the rest of the group finished. Students were asked not to mention the content of the experiment to their classmates after returning to class. Before leaving the room, they were thanked for their effort and participation, and left the room of the experiment together. The experiment took approximately 20 min. After completion of the study, the school was informed about the results by email.

### Results and Discussion

Data were analyzed with SPSS 24. Mean scores for the explicit measures were calculated. For the implicit associations measured by the ST-IAT, the D-scores based on the improved scoring algorithm as described in Greenwald et al. (2003) were calculated. A higher ST-IAT D-score indicates more positive associations with smoking. For all means and standard deviation, see Table 2.

**Table 2:** Means, standard deviation for all dependent variables of Study 1b.

	Risk perception	Explicit attitude	Implicit associations
Social warning condition	*M* = 3.46, *SD* = 1.51	*M* = 2.06, *SD* = 1.00	*M* = –0.35, *SD* = 0.35
Health warning condition	*M* = 3.18, *SD* = 1.57	*M* = 2.07, *SD* = 1.07	*M* = –0.33, *SD* = 0.47
Control condition	*M* = 3.04, *SD* = 1.39	*M* = 1.81, *SD* = 1.02	*M* = –0.30, *SD* = 0.36
Overall	*M* = 3.24, *SD* = 1.49	*M* = 1.98, *SD* = 1.00	*M* = –0.33, *SD* = 0.39

#### Risk Perception and Attitude Toward Smoking

A MANOVA with warning label condition (health warning label vs. social warning label vs. control) as between-subjects factor, and the mean scores of the explicit attitude toward smoking and risk perception as dependent variables showed no significant differences between the three warning label conditions, *F*(2, 87)_risk perception_ = 0.640, *p*_risk perception_ = 0.530 $\eta_{p}^{2}$_risk perception_ = 0.015; *F*(2, 87)_attitude toward smoking_ = 0.622, *p*_attitude toward smoking_ = 0.539, $\eta_{p}^{2}$_attitude toward smoking_ = 0.014.

#### Implicit Associations

An ANOVA with warning label condition (health warning label vs. social warning label vs. control) as between-subjects factor, and the D-score as dependent variable showed no significant differences between the three warning label conditions, *F*(2, 87) = 0.150, *p* = 0.861, $\eta_{p}^{2}$ = 0.004.

Interestingly, no differences between warning label conditions were found in the younger group on risk perception or explicit attitudes toward smoking. Similar to the adult group, effects for implicit associations did not differ significantly between warning label conditions. These results are not in line with earlier research which has shown that social warning labels are more effective in changing explicit cognitions and implicit associations in an adult group (Glock et al., 2013a; Süssenbach et al., 2013). To validate the present findings, in the following two studies we tried to replicate our results of the first two studies, again by testing the paradigm in groups of adult smokers, adult non-smokers, and teenagers. The same explicit measures were assessed, while the ST-IAT was slightly adjusted to measure the associations between smoking and positivity/negativity instead of healthiness/unhealthiness. By doing so, we tried to clarify whether the null findings in the first two studies are due to the fact that associations between smoking and healthiness/unhealthiness are not influenced, while more overall associations between smoking and positivity/negativity are. Additionally, we increased the sample size in order to increase the statistical power (as both Study 1a and Study 1b were underpowered), and tested in a pre-test whether the social warning labels were indeed perceived as less threatening.

## Study 2A

### Methods

#### Participants and Design

Two-hundred-forty-six adults (123 smokers and 123 non-smokers) participated in this study. Four participants were excluded from this sample because they did not provide any information about their smoking status. Thus, the final sample consisted of 242 participants (130 males, 103 females, 1 other, 8 missing values on gender), with an age range between 18 and 67 years old (*M* = 33.73, *SD* = 11.27). A 3 (warning label condition: health warning label vs. social warning label vs. control) × 2 (group: smokers vs. non-smokers) between-subjects design was used, with risk perception, explicit attitude toward smoking, and implicit associations toward smoking as dependent variables. Participants were randomly assigned to one of the warning label conditions. Data were acquired online using Inquisit 4 (Inquisit computer software, 2015). Distribution to conditions was as follows: 85 participants in the health warning label condition (41 smokers, 44 non-smokers), 82 participants in the social warning label condition (44 smokers, 38 non-smokers), and 75 participants in the control condition (38 smokers, 37 non-smokers). This study was disseminated using Prolific: participants signed up for the study and received a link to download the study file onto their computer. Before participants could start with the experiment, they were asked to ensure that they would not be interrupted for the duration of the experiment, and had to give active consent. The experiment took approximately 20 min, and participants received 1.7 pounds as compensation. The study was conducted according to the principles expressed in the Declarations of Helsinki and was approved by the ethical committee of the Faculty of Social Sciences at Radboud University (ECWS-2017-061). Data collection took place one year after Study 1a and Study 1b.

#### Procedure and Materials

The same procedure, materials, and measures were used as in Study 1a, and all materials were translated from Dutch to English. Reliability for the St-Iat and questionnaires were as follows: Iat (Guttman split-half = 0.67), risk perception (Cronbach’s α = 0.92), and attitudes toward smoking (Cronbach’s α = 0.88). Social and health warning labels were tested in a pilot study in a group of 53 non-smoking students (27 males, 25 females) who rated each warning label on whether the text matches the image, whether the label elicits negative emotions, whether the label shows strong social consequences, how threatening the label is, how aversive the label is, and whether participants agree with the content of the label. All answers were given on a 7-point Likert scale. Results showed that on all variables health warnings were rated higher than social warning labels, all p’s < 0.001. In addition, the following changes were performed: During the St-Iat, participants had to categorize upcoming images or words as positive or negative by pressing the “E” and the “I” keys as quickly as possible, without making too many errors. Instead of healthy and unhealthy images, positive words (e.g., excellent, fabulous) and negative words (e.g., stupid, painful) were displayed. After completion of the task, participants were thanked, paid, and debriefed.

### Results and Discussion

Data were analyzed with SPSS 24, and mean scores for risk perception and attitude were calculated. For the implicit associations measured by the ST-IAT, D-scores based on the improved scoring algorithm as described in Greenwald et al. (2003) were calculated. A higher ST-IAT D-score indicates more positive associations with smoking. Six participants were excluded from the analyses because they made too many errors during the implicit association task (>10% of the trials). Furthermore, three participants were excluded because their ST-IAT scores were 2.5SDs above or below the group means. For all means and standard deviation, see Table 3.

**Table 3:** Means, standard deviation for all dependent variables of Study 2a.

		Risk perception	Explicit attitude	Implicit associations
Non-smokers	Social warning condition	*M* = 3.78, *SD* = 1.88	*M* = 2.28, *SD* = 1.18	*M* = –0.39, *SD* = 0.30
	Health warning condition	*M* = 3.56, *SD* = 1.46	*M* = 2.31, *SD* = 1.19	*M* = –0.35, *SD* = 0.48
	Control condition	*M* = 4.01, *SD* = 1.43	*M* = 2.38, *SD* = 1.18	*M* = –0.21, *SD* = 0.61
	Overall	*M* = 3.77, *SD* = 1.59	*M* = 2.32, *SD* = 1.18	*M* = –0.19, *SD* = 0.58
Smokers	Social warning condition	*M* = 4.73, *SD* = 1.35	*M* = 3.64, *SD* = 1.15	*M* = –0.31, *SD* = 0.68
	Health warning condition	*M* = 4.66, *SD* = 1.57	*M* = 3.85, *SD* = 1.21	*M* = –0.04, *SD* = 0.61
	Control condition	*M* = 3.90, *SD* = 1.44	*M* = 3.43, *SD* = 1.16	*M* = –0.04, *SD* = 0.60
	Overall	*M* = 4.46, *SD* = 1.48	*M* = 3.64, *SD* = 1.18	*M* = –0.11, *SD* = 0.65

#### Risk Perception and Attitude Toward Smoking

**FIGURE 1:**
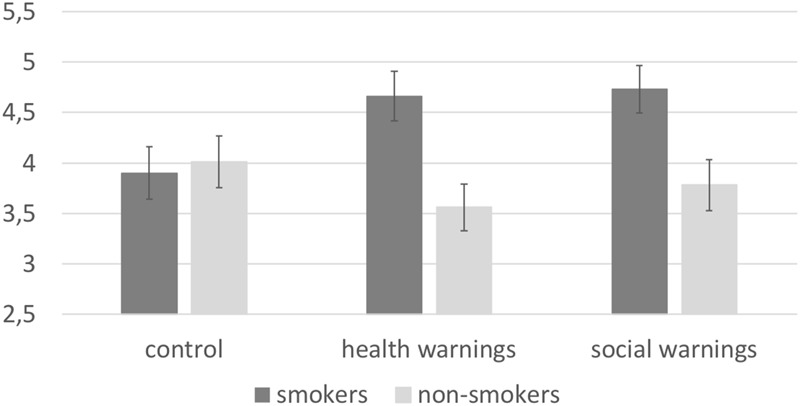
Risk perception toward smoking, as a function of group (smokers vs. non-smokers) and condition (health warnings vs. social warnings vs. control). Error bars represent standard errors.

A MANOVA with warning label condition (health warning label vs. social warning label vs. control) and group (smokers vs. non-smokers) as between-subjects factor, and the mean scores of the explicit attitude toward smoking and risk perception as dependent variables was conducted. For risk perception, the main effect of warning label condition was not significant, *F*(2, 227) = 0.705, *p* = 0.495, $\eta_{p}^{2}$ = 0.006. However, a significant main effect of group was found, *F*(1, 227) = 10.413, *p* = 0.001, $\eta_{p}^{2}$ = 0.044, with smokers scoring higher on risk perception (*M* = 4.46, *SD* = 1.48) than non-smokers (*M* = 3.77, *SD* = 1.59). The interaction between warning label condition and group was significant, *F*(2, 227) = 3.517, *p* = 0.031, $\eta_{p}^{2}$ = 0.030 (see Figure 1). Simple effect analyses revealed that smokers in the control condition seem to have a lower risk perception (*M* = 3.90, *SD* = 1.44) than smokers in the social warning label condition (*M* = 4.72, *SD* = 1.35, *p* = 0.053). The comparison between smokers in the control condition and smokers in the health warning label condition (*M* = 4.66, *SD* = 1.57) did not reach significance (*p* = 0.09, *d* = 0.50), all other comparisons non-significant).

For attitude, no significant effect of warning label condition was found, *F*(2, 227) = 0.443, *p* = 0.643, $\eta_{p}^{2}$ = 0.004. However, a significant main effect of group was found, *F*(1, 227) = 72.098, *p <* 0.001, $\eta_{p}^{2}$ = 0.241, with smokers having a more positive attitude toward smoking (*M* = 3.65, *SD* = 1.18) than non-smokers (*M* = 2.32, *SD* = 1.18). The interaction between warning label condition and group was not significant, *F*(2, 227) = 0.851, *p* = 0.428, $\eta_{p}^{2}$ = 0.007.

#### Implicit Associations

**FIGURE 2:**
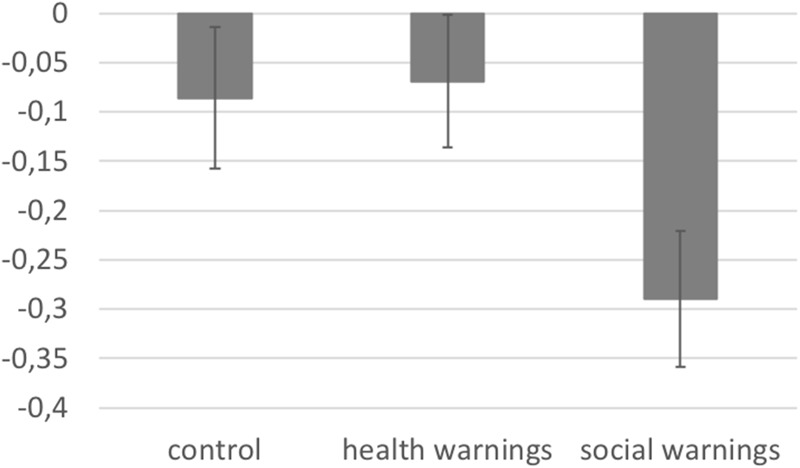
D-scores as a function condition (health warnings vs. social warnings vs. control). The higher the score, the more positive the implicit associations toward smoking. Error bars represent standard errors.

An ANOVA with warning label condition (health warning label vs. social warning label vs. control) and group (smokers vs. non-smokers) as between-subjects factors, and the D-score as dependent variable showed significant differences between the three warning label conditions, *F*(2, 227) = 3.153, *p* = 0.045, $\eta_{p}^{2}$ = 0.027 (see Figure 2). *Post hoc* analyses revealed that participants in the health warning label condition scored marginally more positive on implicit associations toward smoking (*M* = –0.071, *SD* = 0.58) compared to participants in the social warning label condition (*M* = –0.29, *SD* = 0.64, *p* = 0.069, *d* = 0.36). The remaining comparisons were non-significant (health warnings condition vs. control condition *p* = 1.00, *d* = 0.03; social warnings conditions vs. control condition *p* = 0.13, *d* = 0.32). No significant differences between the smoker and non-smoker groups were found, *F*(1, 227) = 1.377, *p* = 0.242, $\eta_{p}^{2}$ = 0.006, and the interaction between warning label condition and group was also not significant, *F*(2, 227) = 1.101, *p* = 0.334, $\eta_{p}^{2}$ = 0.010.

Consistently with Study 1a, our results show that smokers have a higher risk-perception and a more positive attitude toward smoking than non-smokers. No significant main effect of warning label condition was found, thus the finding of Study 1a that participants who watched the health warnings scored higher on risk-perception than participants who watched the social warnings was not replicated. However, smokers who were presented with social warning labels had a higher risk perception than smokers in the control condition. Comparable numerical effects were found for health warning labels, but it did not reach significance. This suggests that at least for smokers, warning labels are effective in altering explicit cognitions. Importantly, the social warning labels also led to more negative implicit associations toward smoking compared to the health warning labels, irrespectively of smoking status. This is in line with our expectations that the negative social consequences depicted on the labels could activate associations between smoking and negativity. Participants in the control condition who did not see any warning labels had comparable implicit association scores as those in the health warning label condition, which supports this assumption.

## Study 2B

### Methods

#### Participants and Design

One-hundred-eleven students from two Dutch secondary schools participated in this study. From this sample, 16 students (7 participants from the health warning label condition, 2 participants from the social label condition, and 7 participants from the control condition) were excluded because they smoked. Thus, the final sample consisted of 95 students (49 males, 42 females, 4 missing values on gender) with an age range between 16 and 17 years old (*M* = 16.33, *SD* = 0.47). All students were studying at VWO level. Distribution to conditions was as follows: 28 participants in the health warning label condition, 36 participants in the social warning label condition, and 31 participants in the control condition. As in Study 1b, active informed consent was acquired from the director of the school, and subsequently, passive informed consent was acquired from the parents or primary caretakers of the participating students. Before participating in the study, all students gave active written informed consent by themselves.

A single factor (warning label condition: health warning label vs. social warning label vs. control) between-subject design was used, with risk perception, explicit attitude toward smoking, and implicit associations toward smoking as dependent variables. Participants were randomly assigned to one of the conditions using Inquisit 4 (Inquisit computer software, 2015). The study was conducted according to the principles expressed in the Declarations of Helsinki and was approved by the ethical committee of the Faculty of Social Sciences at Radboud University (ECWS-2017-061). Data collection took place one year after Study 1a and Study 1b.

#### Procedure and Materials

The procedure was similar to the procedure of Study 1b: On the days of the experiment, students were picked up from their classes in groups of 6 students. They received verbal instructions about the experiment and read and signed the active informed consent. The same materials and measures were used as in Study 2a. Participants first performed the ST-IAT (Guttman split-half = 0.57), and subsequently the questionnaires measuring smoking-related risk perception (Cronbach’s α = 0.87) and attitudes toward smoking (Cronbach’s α = 0.88). The last part of the questionnaire contained demographic questions asking about gender, age, grade, school level, their smoking status, and smoking habits. After completion, students waited until the rest of the group finished. Before leaving the room, they were thanked for their effort and participation, and left the room of the experiment together. The experiment took approximately 20 min. After completion of the study, the school received the results via email.

### Results and Discussion

Data were analyzed with SPSS 24, and mean scores for risk perception and attitude were calculated. For the implicit associations measured by the ST-IAT, D-scores based on the improved scoring algorithm as described in Ganster et al. (1983) were calculated. A higher ST-IAT D-score indicates more positive associations with smoking. One participant was excluded because the ST-IAT scores were 2.5SDs above or below the group means. For all means and standard deviation, see Table 4.

**Table 4:** Means, standard deviation for all dependent variables of Study 2b.

	Risk perception	Explicit attitude	Implicit associations
Social warning condition	*M* = 2.83, *SD* = 1.32	*M* = 2.42, *SD* = 1.13	*M* = –0.13, *SD* = 0.48
Health warning condition	*M* = 2.66, *SD* = 1.29	*M* = 1.49, *SD* = 0.68	*M* = –0.09, *SD* = 0.59
Control condition	*M* = 2.86, *SD* = 1.33	*M* = 1.93, *SD* = 1.02	*M* = –0.29, *SD* = 0.68
Overall	*M* = 2.79, *SD* = 1.30	*M* = 1.99, *SD* = 1.04	*M* = –0.17, *SD* = 0.58

#### Risk Perception and Attitude Toward Smoking

A MANOVA with warning label condition (health warning label vs. social warning label vs. control) as between-subjects factor, and the mean scores of the explicit attitude toward smoking and risk perception as dependent variables was conducted. The main effect of warning label condition was not significant for risk perception, *F*_(2, 91)_ = 0.038, *p* = 0.826, $\eta_{p}^{2}$ = 0.004, and significant for attitude towards smoking, *F*_(2, 91)_ = 6.987, *p* = 0.002 $\eta_{p}^{2}$ = 0.133. Participants in the social warning label condition had a more positive attitude towards smoking (*M* = 2.42, *SD* = 1.13) than participants in the health warning label condition (*M* = 1.49, *SD* = 0.68; *p* = 0.001, *d* = 0.99). All other comparisons were non-significant (health warnings condition vs. control condition *p* = 0.259, *d* = 0.51; social warnings conditions vs. control condition *p* = 0.134, *d* = 0.46).

#### Implicit Associations

An ANOVA with warning label condition (health warning label vs. social warning label vs. control) as between-subjects factor, and the D-score as dependent variable showed no significant differences between the three warning label conditions, *F*_(2, 91)_ = 0.889, *p* = 0.415, $\eta_{p}^{2}$ = 0.019.

Again, no significant differences between warning label conditions were found in the younger group on risk perception. Unexpectedly, the social warning label condition elicited more positive attitudes toward smoking than the health warning label condition, which is not in line with previous studies in which the reactance response was found when participants were exposed to health warning labels (Süssenbach et al., 2013). Contrary to the adult group, no significant differences between warning label conditions were found on implicit associations.

## General Discussion

In the current study, we investigated whether warning labels challenging positive social outcome expectancies lead to an improved effectiveness, and thus to a higher risk perception (because of no resistance responses), and more negative explicit attitudes and implicit associations, compared to health warning labels and no warning labels for both teenagers and adults. Our results show an inconsistent pattern for both groups: in the adult group, health warning labels lead to a higher risk perception than social warning labels in Study 1a, while this effect was not significant in Study 2a. Interestingly, a significant interaction between the smoking status of the participants and the type of warning label was significant in Study 2a, suggesting that for smokers both types of warning labels increased risk perception. It is important to note, however, the comparison between the control condition and the health warning condition did not reach significance for smokers, but showed similar numerical differences. Furthermore, as expected, implicit associations were more negative after presentation of social warning labels in Study 2a. Explicit attitudes did not differ significantly depending on warning label type. Consistently across two studies, smokers had a more positive explicit attitude toward smoking, a higher risk perception that they will suffer from smoking-induced health consequences, and stronger compensatory health beliefs (only assessed in Study 1a) than non-smokers. For teenagers, only attitude did significantly differ depending on label type, with social warning labels leading to a more positive explicit attitude in Study 2b.

Our findings for the teenage groups are not in line with prior expectancies that social warning labels would lead to a higher risk perception, more negative attitudes toward smoking, and stronger implicit associations between smoking and negativity. Teenagers are very sensitive to negative social consequences and peer pressure (Glock et al., 2013a; Liao et al., 2013). And recent research shows that young adults perceive negative social consequences as very aversive (Albers and Lakens, 2018). Therefore, we assumed that warning labels challenging positive social outcome expectancies (Glock et al., 2013b; Süssenbach et al., 2013) could have positive effects in this younger group. However, only in Study 2b, a weak effect of warning labels was found, with students who saw social warnings having a more positive attitude than students who saw health warning labels. This could be interpreted as a possible reactance response. Given the small effect size and the fact that no such effect was found in Study 1b, this finding should be interpreted with caution and first be replicated before clear explanations can be given.

Results of the adult samples first show that, not surprisingly, smokers have a more explicit positive attitude toward smoking, a higher risk perception, and higher compensatory health beliefs (only assessed in Study 1a) compared to non-smokers, which is in line with previous work (Glock et al., 2013a). Looking at the effects of warning labels, a significant interaction between smoking status and warning label on risk perception was found in Study 2a. Being exposed to social warning labels lead to a significant higher risk perception in smokers, suggesting that warning labels are a useful tool to influence explicit cognitions of smokers and could lead to smoking cessation on the long run. Furthermore, for health warning labels, numerical differences pointed to a comparable effect, which was, however, not significant. Although this could be interpreted as a first support for using social warning labels and even health warning labels to convince smokers to stop smoking, given that this effect was not found in Study 1a, future research should replicate this finding before strong conclusions can be drawn. Previous research demonstrates mixed results of graphical warning labels on explicit evaluations, with on the one hand research showing an influence of warning labels on explicit attitudes (Süssenbach et al., 2013), while more recent work (van Dessel et al., 2018) on the other hand showing no beneficial effects of graphical warning labels compared to text-only warning labels on explicit evaluations.

In addition, implicit associations did not differ depending on smoking status, which is not in line with previous work (Sherman et al., 2003; de Houwer et al., 2006; Tibboel et al., 2011; Smith and de Houwer, 2015; but see also Glock et al., 2013a). Interestingly, while no significant influence of warning labels on the association between smoking and unhealthy state was found in Study 1a, social warning labels led to stronger associations between smoking and negativity in general in Study 2a. Important to note, health warning labels did not differ significantly from the control condition, suggesting that the effect is driven by an influence of the social warning labels which lead to a more negative response. Previous research did not find an influence of warning label on implicit associations (Süssenbach et al., 2013; van Dessel et al., 2018), and given that our effect size is rather small, results should be interpreted with caution.

The reason for not detecting an influence of warning labels could lie in the lack of believability of the social warning labels. Although we used warning labels which were shown to be effective and believable in previous research (Glock et al., 2012), our post-test result suggests that social warning labels were perceived as less believable than the health warning labels. Unfortunately, believability of the message was tested in a separate group of students, making it impossible to test whether believability could mediate the effect of warning label condition. It would, therefore, be interesting to take this into account in future research, next to other known mediators such as self-efficacy (e.g., see Kok et al., 2017). In addition, there are several limitations of our research which are important to mention. Firstly, we did not look at behavioral effect which the present warning labels might have, but only investigated their influence on explicit cognitions and implicit associations. This can be problematic because research has shown that often, the attitude-behavior link is rather weak (Glock et al., 2013c). Therefore, the use of behavioral measures is highly recommended by different scholars in the field (e.g., Kok et al., 2017) in order to be able to make clear predictions about the effectiveness of different kinds of warning labels. Future research should investigate possible changes in real smoking behavior. Secondly, although we were able to use a diverse adult sample in Study 2a to increase generalizability, our teenage sample consisted only of highly educated students. In addition, teenagers who already smoked were excluded. For future research, it would be important to use a more diverse sample consisted of both higher and lower educated teenagers, and look at whether effects on teenager smokers differ and are comparable to adult smokers. Thirdly, several warning labels were presented directly after each other and the measurements were taken directly after the presentation of the labels. Additionally, participants were only exposed to the warning labels once. All three points lead to a decrease in the research ecological validity. Therefore, it is recommended to use a more ecological valid design and try to assess long-term influences (see for example Gendall et al., 2017) in order to clarify possible habituation effects, but also behavioral outcomes such as whether and when non-smoking teenagers start to smoke later in life. Fourthly, for all four studies, the sample was rather small, especially for assessing significant effects for implicit associations. Although we performed an *a priori* power analysis, it can be that this analysis was biased as it was not based on a meta-analysis (see also Albers and Lakens, 2018). Furthermore, prior research observed rather small effect sizes with much larger samples (Macy et al., 2015), and together, future research is needed to clarify whether the non-significant results in the present study reflect a non-existing effect or whether our actual effect size was too small to be detected with a possibly biased sample size.

The goal of the current study was to investigate how warning labels can be made more effective and to clarify whether and how the content of the warning labels influences teenagers’ implicit associations and explicit cognitions toward smoking. As most people start to smoke early in life, it is essential to investigate different ways to prevent teenagers from starting to smoke in the first place. Our findings are a first step to help find new ways to reach this goal and motivate future research to focus on how warning labels should be designed to be more effective.

## Author Contributions

BM developed the research questions, designed the experiments, supervised the data collection, and wrote the manuscript. RH, SK, and HY developed the research questions, designed the experiments, collected the data at the schools, and wrote the manuscript. SL collected the data of the adult groups and wrote the manuscript.

## Conflict of Interest Statement

The authors declare that the research was conducted in the absence of any commercial or financial relationships that could be construed as a potential conflict of interest.

## Footnotes

1 All materials, data, and syntax can be received from the first author upon request.

2 We applied this power-analysis to all four studies.

3 Due to a programming error, the compensatory health beliefs questionnaire was not assessed in the subsequent studies.
