# Corrigendum: Social Tobacco Warnings Can Influence Implicit Associations and Explicit Cognitions

**DOI:** 10.3389/fpsyg.2019.01408

**Published:** 2019-06-24

**Authors:** Barbara C. N. Müller, Rinske Haverkamp, Silvia Kanters, Huriye Yaldiz, Shuang Li

**Affiliations:** ^1^Behavioural Science Institute, Radboud University, Nijmegen, Netherlands; ^2^Radboud UMC, Nijmegen, Netherlands

**Keywords:** tobacco warning labels, smoking, risk perception, attitudes toward smoking, implicit associations

In the original article, there was an error.

Due to a wrong filter used, incorrect data was reported in Study 2b. This made it necessary to adjust the demographics, reported distribution of participants across conditions, reliabilities of scales, and results.

A correction has been made to **Study 2b**, **Methods, paragraph 1** and **paragraph 3; Results and discussion; paragraph 1, 2, and 3** and Table 4.

**Table 4:** Means, standard deviation for all dependent variables of Study 2b.

	**Risk perception**	**Explicit attitude**	**Implicit associations**
Social warning condition	*M* = 2.83, *SD* = 1.32	*M* = 2.42, *SD* = 1.13	*M* = −0.13, *SD* = 0.48
Health warning condition	*M* = 2.66, *SD* = 1.29	*M* = 1.49, *SD* = 0.68	*M* = −0.09, *SD* = 0.59
Control condition	*M* = 2.86, *SD* = 1.33	*M* = 1.93, *SD* = 1.02	*M* = −0.29, *SD* = 0.68
Overall	*M* = 2.79, *SD* = 1.30	*M* = 1.99, *SD* = 1.04	*M* = −0.17, *SD* = 0.58

## Corrected Paragraph

### Participants and Design

One-hundred-eleven students from two Dutch secondary schools participated in this study. From this sample, 16 students (7 participants from the health warning label condition, 2 participants from the social label condition, and 7 participants from the control condition) were excluded because they smoked. Thus, the final sample consisted of 95 students (49 males, 42 females, 4 missing values on gender) with an age range between 16 and 17 years old (*M* = 16.33, *SD* = 0.47). All students were studying at VWO level. Distribution to conditions was as follows: 28 participants in the health warning label condition, 36 participants in the social warning label condition, and 31 participants in the control condition. As in Study 1b, active informed consent was acquired from the director of the school, and subsequently, passive informed consent was acquired from the parents or primary caretakers of the participating students. Before participating in the study, all students gave active written informed consent by themselves.

### Procedure and Materials

The procedure was similar to the procedure of Study 1b: On the days of the experiment, students were picked up from their classes in groups of 6 students. They received verbal instructions about the experiment and read and signed the active informed consent. The same materials and measures were used as in Study 2a. Participants first performed the ST-IAT (Guttman split-half = 0.57), and subsequently the questionnaires measuring smoking-related risk perception (Cronbach's α = 0.87) and attitudes toward smoking (Cronbach's α = 0.88). The last part of the questionnaire contained demographic questions asking about gender, age, grade, school level, their smoking status, and smoking habits. After completion, students waited until the rest of the group finished. Before leaving the room, they were thanked for their effort and participation, and left the room of the experiment together. The experiment took approximately 20 min. After completion of the study, the school received the results via email.

### Results and Discussion

Data were analyzed with SPSS 24, and mean scores for risk perception and attitude were calculated. For the implicit associations measured by the ST-IAT, D-scores based on the improved scoring algorithm as described in Ganster et al. (1983) were calculated. A higher ST-IAT D-score indicates more positive associations with smoking. One participant was excluded because the ST-IAT scores were 2.5SDs above or below the group means. For all means and standard deviation, see Table 4.

#### Risk Perception and Attitude Toward Smoking

A MANOVA with warning label condition (health warning label vs. social warning label vs. control) as between-subjects factor, and the mean scores of the explicit attitude toward smoking and risk perception as dependent variables was conducted. The main effect of warning label condition was not significant for risk perception, *F*_(2, 91)_ = 0.192, *p* = 0.826, η_p_^2^ = 0.004, and significant for attitude towards smoking, *F*_(2, 91)_ = 6.987, *p* = 0.002 η_p_^2^ = 0.133. Participants in the social warning label condition had a more positive attitude towards smoking (*M* = 2.42, *SD* = 1.13) than participants in the health warning label condition (*M* = 1.49, *SD* = 0.68; *p* = 0.001, *d* = 0.99). All other comparisons were non-significant (health warnings condition vs. control condition *p* = 0.259, *d* = 0.51; social warnings conditions vs. control condition *p* = 0.134, *d* = 0.46).

#### Implicit Associations

An ANOVA with warning label condition (health warning label vs. social warning label vs. control) as between-subjects factor, and the D-score as dependent variable showed no significant differences between the three warning label conditions, *F*_(2, 91)_ = 0.889, *p* = 0.415, η_p_^2^ = 0.019.

The authors apologize for this error and state that this does not change the scientific conclusions of the article in any way. The original article has been updated.

